# Changes in Abundance of Oral Microbiota Associated with Oral Cancer

**DOI:** 10.1371/journal.pone.0098741

**Published:** 2014-06-02

**Authors:** Brian L. Schmidt, Justin Kuczynski, Aditi Bhattacharya, Bing Huey, Patricia M. Corby, Erica L. S. Queiroz, Kira Nightingale, A. Ross Kerr, Mark D. DeLacure, Ratna Veeramachaneni, Adam B. Olshen, Donna G. Albertson, Muy-Teck Teh

**Affiliations:** 1 Bluestone Center for Clinical Research, New York University College of Dentistry, New York, New York, United States of America; 2 Department of Oral and Maxillofacial Surgery, New York University College of Dentistry, New York, New York, United States of America; 3 Bioinformatics Department, Second Genome, San Bruno, California, United States of America; 4 Helen Diller Family Comprehensive Cancer Center, University of California San Francisco, San Francisco, California, United States of America; 5 Department of Oral and Maxillofacial Pathology, Radiology and Medicine, New York University College of Dentistry, New York, New York, United States of America; 6 Departments of Otolaryngology and Plastic Surgery, New York University, New York, New York, United States of America; Barts & The London School of Medicine and Dentistry, Queen Mary University of London, United Kingdom

## Abstract

Individual bacteria and shifts in the composition of the microbiome have been associated with human diseases including cancer. To investigate changes in the microbiome associated with oral cancers, we profiled cancers and anatomically matched contralateral normal tissue from the same patient by sequencing 16S rDNA hypervariable region amplicons. In cancer samples from both a discovery and a subsequent confirmation cohort, abundance of *Firmicutes* (especially *Streptococcus*) and *Actinobacteria* (especially *Rothia*) was significantly decreased relative to contralateral normal samples from the same patient. Significant decreases in abundance of these phyla were observed for pre-cancers, but not when comparing samples from contralateral sites (tongue and floor of mouth) from healthy individuals. Weighted UniFrac principal coordinates analysis based on 12 taxa separated most cancers from other samples with greatest separation of node positive cases. These studies begin to develop a framework for exploiting the oral microbiome for monitoring oral cancer development, progression and recurrence.

## Introduction

Annually, ∼22,000 Americans are diagnosed with oral cancer of which 90% are squamous cell carcinoma (SCC). The five-year survival, at 40% has not improved in the past 40 years, and it is one of the lowest of the major cancer sites, resulting in more people dying from oral cancer than melanoma, cervical or ovarian cancer in the USA. Worldwide there are 350,000–400,000 new cases diagnosed each year. Unlike most other anatomic sites, which have decreased in cancer incidence, the incidence of oral cancer is increasing, particularly among young people and women [1], [2]. The major risk factors, tobacco and alcohol use, cannot explain the changes in incidence, because oral cancer also commonly occurs in patients without a history of tobacco or alcohol exposure [3]. Recently, human papillomavirus (HPV) has been identified as an etiologic agent for oropharyngeal cancer, but HPV infection is not a significant contributor to oral cancer, as the virus is rarely found in these cancers (2–4% of cases) [4]. Thus, contributions from other, possibly environmental factors remain to be found.

A role for bacterial infection in causing or promoting cancer is well known with respect to the association of *Helicobacter pylori* with gastric cancer [5], and other cancers, including gallbladder, colon, lung and prostate, have been associated with particular bacterial infections [6], [7], [8]. It is reasonable to ask, therefore, if shifts in the composition of the normal oral cavity microbiome, comprised of more than 600 different bacterial species [9] and/or chronic bacterial infection could be promoters or causes of oral cancer. Indeed, changes in the microbial community are commonly associated with dental diseases such as periodontal disease, which is most likely a polymicrobial disease characterized by outgrowth of certain pathologic organisms [10], and chronic periodontitis has been reported to be a risk factor for oral premalignant lesions and cancers [11]. Elevated levels and changes in the composition of bacterial and fungal microbiota of the oral cavity have been reported in association with oral pre-cancers and cancers [12]. There is, however, no consensus amongst reports regarding cancer-associated changes in the oral microbiome. This confusion may have arisen because early studies were limited to analysis of the relatively small numbers of known and cultivable oral bacterial species [13], [14], and later studies using molecular methods focused on particular phyla [15] or cloned and sequenced small numbers of clones per sample [16], [17].

Culture independent methods, particularly those employing next generation sequencing of the hypervariable region of the 16S ribosomal subunit, provide a means to more comprehensively and accurately profile the microbiome in health and disease [18]. Such studies of the oral microbiome [16], [19], [20], [21], [22], [23], [24], [25], [26], [27], [28] reveal, on the one hand, that the healthy oral microbiome is characterized by a relatively small number of bacterial phyla (9–13), the most commonly reported abundant phyla being *Firmicutes*, *Proteobacteria*, *Bacteroidetes*, *Actinobacteria*, and *Fusobacteria* [16], [19], [20], [21], [22], [23], [24], [25], [26], [27], [28]. On the other hand, the majority of inter-individual variation has been attributed to diversity at the species or strain level [24]. *Streptococcus* is most often observed to be the dominant genus in the healthy oral microbiome, and less frequently *Prevotella*, *Veillonella*, *Neisseria*, and *Haemophilus* dominate an individual's oral microbiome [19], [24]. Variation is also observed in the microbial community composition of biofilms at each intraoral habitat (*e.g*., tooth surface, lateral and dorsal tongue, etc.), most likely reflecting the different surface properties and microenvironments [21], [24].

To properly investigate possible shifts in the composition of the oral microbiome in oral cancer, therefore, it is necessary to control for differences between oral subsites and inter-individual variation. In addition, high recurrence rates and prevalence of second primary oral cancers support the proposal that these cancers develop out of a field of genetically altered cells, the concept of “field cancerization” [29]. Such fields have been reported to extend as much as 7 cm from a tumor and to appear clinically normal [30]. For these reasons, we investigated the oral cancer associated microbiome by non-invasively sampling the cancer lesion and an anatomically matched contralateral region of normal tissue from each individual. We subjected DNA isolated from these samples to 16S ribosomal subunit amplification and sequencing. The aim of these studies was to begin to lay a foundation that would allow exploitation of the oral microbiome for treatment and monitoring of oral cancer initiation, progression and recurrence.

## Results

To investigate changes in the oral microbiome associated with oral cancer, we prospectively collected cancer and anatomically matched patient clinically normal samples from five patients (Table 1, Study 1 Discovery Cohort, Table S1). To confirm and extend our initial observations, we performed a second study (Study 2, Tables S2 – S6, total number of samples  = 83) in which we prospectively collected an independent set of lesional and anatomically matched clinically normal samples from oral cancer (Table 2, Study 2 Confirmation Cohort, Table S2), carcinoma *in situ* (CIS, Table S3) and pre-cancer patients (Table S4), as well as from the left and right sides of the lateral tongue and floor of mouth of healthy normal individuals (Table S5). In Study 2, we also included an independent analysis of the initial five cancer patients from Study 1 and six pairs of replicate samples (three cancer and three pre-cancer patients, Table S6). The latter were included to assess reproducibility of sample collection and processing and were not included in any of the analyses (see further discussion in Methods).

**Table 1:** Discovery Cohort Cancer Patient Characteristics.

Patient	Age	Sex	Site	Tumor size (cm)	pTNM^1^	Immunocompromised	Smoker	Alcohol
113	62	M	Right buccal mucosa	2.5×3.0	pT2N2bMx	Yes	Previous	Current
116	84	F	Right retromolar trigone	6.0	pT4aN0Mx	No	Previous	Previous
117	68	M	Left posterior lateral tongue	1.5×1.0	pT2N0Mx	No	Previous	Current
136	68	M	Left lateral tongue	5.0×2.0	pT2N2bMx	No	Never	Current
142	64	M	Right dorsum of tongue	0.3×1.5	pT3N0M0	Yes	Current	Current

1 Pathologic tumor stage according to the American Joint Committee on Cancer guidelines (T  =  tumor size, N  =  regional lymph node metastasis, M =  distant metastasis)

**Table 2:** Conformation Cohort Cancer Patient Characteristics.

Patient	Age	Sex	Site	Tumor size (cm)	pTNM^1^	Immunocompromised	Smoker	Alcohol
104	55	F	Right lateral tongue	2.5×2.5	pT4N2M0	No	Current	Current
108	39	F	Right lateral tongue corner	3.0	pT4N2bM0	No	Previous	Non-drinker
114	48	M	Left post. mandibular gingiva	1.5	pT1N0M0	Yes	Non-smoker	Current
114	48	M	Left post. maxillary gingiva	2.5	pT2N0M0	Yes	Non-smoker	Current
128	71	M	Left alveolar ridge	4.0×3.0	pT4bN0M0	No	Previous	Previous
143	61	M	Right floor of mouth	1.6×1.0	pT1N0M0	No	Current	Current
144	70	M	Left floor of mouth	3.5×1.5	pT1N0M0	No	Previous	Current
146	78	M	Right retromolar trigone	1.5×1.5	pT1N2bMx	No	Previous	Current
153	51	F	Left ventral tongue	8.0×8.0	pT2N1Mx	No	Non-smoker	Current
156	69	F	Right mandibular gingiva	1.0×1.0	pT1N0M0	No	Non-smoker	Current

1 Pathologic tumor stage according to the American Joint Committee on Cancer guidelines (T  =  tumor size, N  =  regional lymph node metastasis, M  =  distant metastasis

### Study 1. Discovery cohort

We swabbed the oral cancer lesion and a corresponding anatomically matched clinically normal tissue area from the Discovery Cohort of five patients (Table 1). Using the Roche GS Junior instrument to perform pyrosequencing, we obtained, in a single run, a total of 104,380 sequences from amplicons that spanned the V4 hypervariable region of the bacterial 16S small ribosomal subunit (Table S7). The number of raw sequence reads varied by >10-fold across samples, ranging from 1,231 to a maximum of 17,682 raw reads. Quality filtered sequences were searched against the Greengenes reference database of 16S sequences, clustered at 97%, and Operational Taxonomic Units (OTUs) were assigned taxonomic classification using mothur's Bayesian classifier. Of the 92,987 sequences that passed quality filtering, 81,308 were similar to known bacteria and most could be classified to the genus level (65,037) with fewer classified at the species level (17,115). Sequence coverage was variable across samples; the number of reads per sample assigned to OTUs (excluding those filtered due to poor quality or lack of a related sequence in the Greengenes reference database) ranged from 1,038 to a maximum of 14,359, and comprised 76–85% of raw sequences (Table S7). A total of 276 OTUs were identified (per sample range, 37–161, (Table S8). Rarefaction analysis performed at the family level demonstrated a fairly wide range of α diversity with the number of detected families ranging from ∼15–28 (Figure S1a). Three patient samples, normal and cancer from patient 117 and the normal sample from patient 142, plateaued at fewer families than the other samples, indicating somewhat reduced diversity in these samples. All samples plateaued to some extent, though not entirely; further sequencing would likely reveal additional families. On the other hand, pyrosequencing noise and PCR errors could erroneously increase OTU numbers [31]. A similar effect was seen at the genus level (Figure S1b).

### Diversity of microbiomes associated with anatomically matched oral cancer and normal samples

**Figure 1:**
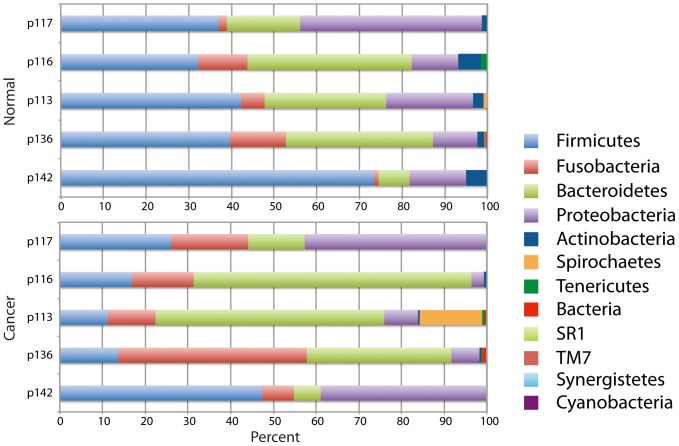
Distribution of phyla in normal and cancer samples in Study 1. The relative distribution of phyla (percent of sequences) is shown for each patient sample with clinically normal samples shown together on top and cancer samples on the bottom.

**Figure 2:**
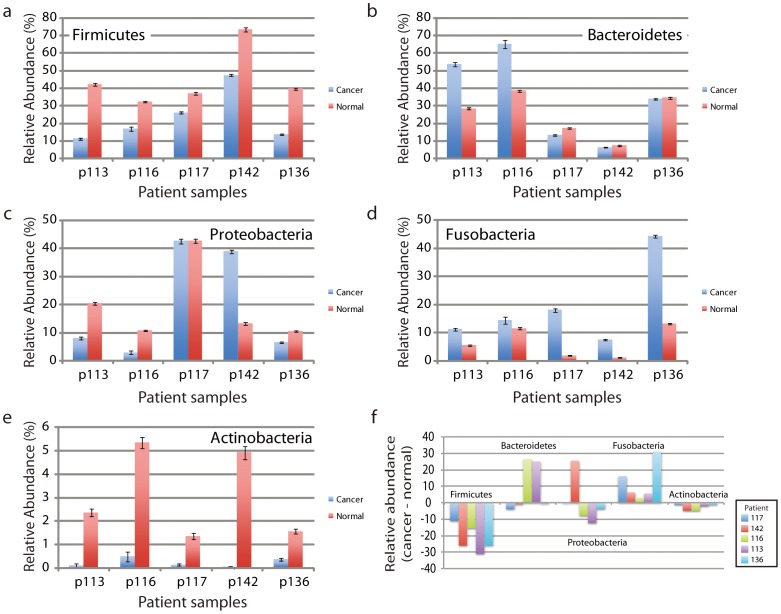
Change in relative abundance of phyla associated with cancer compared to anatomically matched contralateral clinically normal samples in Study 1. (a – e) Relative abundance of each of the five more abundant phyla in cancers compared to clinically normal samples from each of five patients. Note, that data are shown on different scales, reflecting the abundance of the phyla. The magnitudes of the changes in abundance are clearly greater than the statistical counting noise, as indicated by the error bar estimates, which are based on the square root of the actual number of reads. (f) Change in relative abundance shown as the difference in abundance of phyla associated with cancers compared to anatomically matched contralateral clinically normal samples. In cancers, decreases in the relative abundance of *Firmicutes* and *Actinobacteria* were seen in all patients, while the relative abundance of *Fusobacteria* was elevated in cancers from all patients.

The OTUs of the cancer and clinically normal samples in the Discovery cohort were classified into 12 phyla (Table S7). The majority belonged to one of five phyla (99.2%, normal, 98.0% cancers) with the more abundant phyla being *Firmicutes*, *Bacteroidetes*, *Proteobacteria*, *Fusobacteria*, and *Actinobacteria* (Table 7, Figure 1). Although the distribution in cancer and clinically normal samples of these five common phyla varied amongst individuals (Figure 1), in all patients, we observed a significant reduction in the abundance of *Firmicutes* and *Actinobacteria* in cancer compared to the anatomically matched contralateral clinically normal patient sample, (p = 0.004, FDR adjusted p = 0.02 and p = 0.028, FDR adjusted p = 0.07, respectively). We also observed that the proportion of *Fusobacteria* was increased in all patients, but the change in abundance did not reach statistical significance (p = 0.074, Figure 2). We note, however, that to observe consistent changes in abundance of the three phyla in this small cohort is highly significant. Ignoring interactions among the relative abundances of different phyla, a binomial test yields p = 0.0022 as the probability (under the null hypothesis) of having three or more phyla where (all five patients share an increase) or (all five share a decrease).

### Study 2. Confirmation cohort

To confirm these initial observations, we performed Study 2. As before, we swabbed both the lesion (cancer or pre-cancer) and a contralateral anatomically clinically normal matched site. We swabbed the left and right sides of the lateral tongue and floor of mouth of the healthy individuals. Using the Illumina MiSeq instrument, we sequenced amplicons spanning the 16S rDNA V4 hypervariable region. We obtained 4,486,196 raw sequence reads with a range of 31,109 to 125,847 reads per sample after excluding two samples that failed in sequencing (Tables S9 – S13). We assigned taxonomic classification to the OTUs as before. Of the sequences that passed quality filtering, 4,444,432 were similar to known bacteria and most could be classified to the genus level (4,148,785) with fewer classified at the species level (1,650,037). We identified a total of 2,107 OTUs (per sample range, 90–482, Tables S9 – S13). Rarefaction analysis demonstrated a fairly wide range of α diversity with almost all samples plateauing to some extent (Figure S2). As discussed above, sequencing noise and PCR errors may increase OTU numbers [32].

### Diversity of microbiomes associated with anatomically matched samples from oral cancer, pre-cancer and healthy normal individuals in Study 2

**Figure 3:**
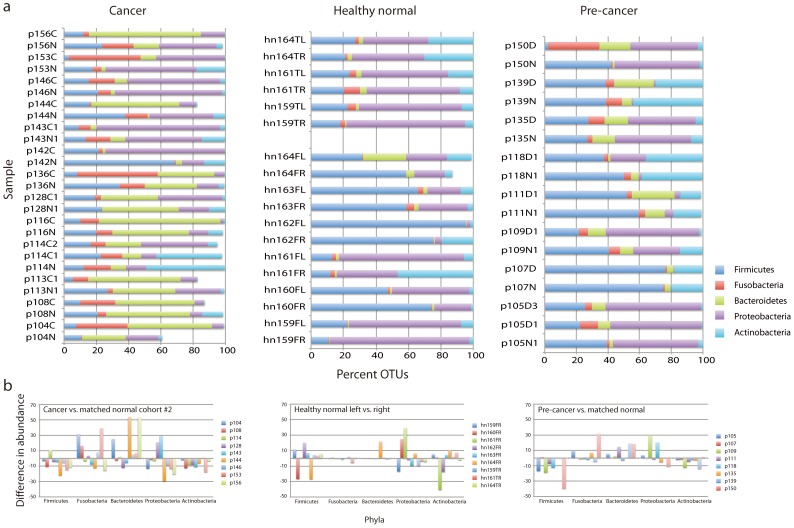
Distribution of phyla in cancer, pre-cancer and healthy normal samples in Study 2. (a) Shown is the relative distribution of phyla (percent of sequences). For cancers, we included only patients for which both the cancer and contralateral clinically normal samples were available. (b) Change in relative abundance of phyla shown as the difference in abundance of phyla associated with cancers or pre-cancers compared to anatomically matched contralateral clinically normal samples. For healthy normal samples, we compared left and right sides of the lateral tongue or floor of mouth.

We first determined that data collected on nine of the 10 Discovery Cohort samples that were successfully profiled in Study 2 (Table S9) were highly correlated with the original data obtained by 454 pyrosequencing (Figure S3). We then considered the Confirmation Cohort comprised only of samples from cancer patients not included in the Discovery Cohort, *i.e*., we excluded the nine Discovery cohort samples (Table 2, Table S9). We again found that the majority of OTUs (61–100%) belonged to one of the five more abundant phyla (*Firmicutes*, *Bacteroidetes*, *Proteobacteria*, *Fusobacteria*, and *Actinobacteria*) (Figure 3a, Table S14). We then asked if there were significant reductions in the abundance of *Firmicutes* and *Actinobacteria* in cancer compared to the anatomically matched contralateral clinically normal patient sample. Indeed, as we had observed in the Discovery cohort (Study 1), there was a significant reduction in abundance of these two phyla in cancers compared to the anatomically matched contralateral clinically normal patient samples (p = 0.042 and p = 0.004, respectively), confirming our initial observations (Figure 3b, Figure S4).

In Study 2, we also observed that the majority of OTUs in samples from pre-cancer patients and healthy normals (Figure 3a) belonged to one of the five phyla *Firmicutes*, *Bacteroidetes*, *Proteobacteria*, *Fusobacteria*, and *Actinobacteria* (pre-cancers  = 99–100%, healthy normal  = 87–100%). We again asked if significant reductions in abundance of *Firmicutes* and *Actinobacteria* might also be present when comparing pre-cancers and anatomically matched contralateral clinically normal patient samples. We did find that abundance of *Firmicutes* and *Actinobacteria* was reduced (p = 0.048 and p = 0.037, respectively), suggesting that changes in abundance of these two phyla may occur early (Figure 3b). By contrast, we did not find significant differences in abundance of these two phyla or any of the five more abundant phyla when comparing the left and right sides of the lateral tongue and floor of mouth of healthy normal individuals (Figure 3b).

To investigate changes in abundance at the genus level for the five more abundant phyla, we considered patient matched samples from the Confirmation Cohort and also included four of the five Discovery Cohort cases that were successfully analyzed in Study 2 (14 cancers from 13 patients, Table S9). We normalized the number of OTUs to one million counts, (Table S15) and for each phylum, we determined changes in abundance of the genera that represented >10% of OTUs in more than 20% of samples (Figure S5 and S6). Significant reduction in abundance was observed for *Streptococcus* (p = 0.003) and *Rothia* (p = 0.021) in cancers relative to anatomically matched clinically normal samples (Table S16). By contrast, we observed increased abundance of *Fusobacterium* (p = 0.044) relative to matched clinically normal samples from the cancer patients. In pre-cancers, we observed significantly reduced abundance of *Streptococcus* (p = 0.042) (Table S16). We found no significant differences in abundance of these more common genera when comparing abundance in samples taken from the left and right sides of the lateral tongue and floor of mouth of healthy individuals (Table S16).

We also noted that although we found no consistent changes in abundance of *Bacteroidetes* when comparing within individuals (Figure 3b), samples from cancer and pre-cancer patients (both lesion and anatomically matched contralateral clinically normal tissue) were associated with increased abundance of *Bacteroidetes* compared to samples from healthy normal individuals (Figure 3a). *Prevotella* species, in particular, differed and included, for example, OTUs corresponding to *P. intermedia*, *P. melaninogenica*, *P. nanceiensis*, *P. oris*, *P. tannerae* and unclassified species (Figure S5, Table S15). Elevated levels of *P. melaninogenica* have been reported previously as a potential salivary biomarker of oral cancer [33] and *P. intermedia* is a periodontal pathogen [34]. Further studies will be required to understand the contributions of general (*Bacteroidetes*) and lesion-specific changes (*Actinobacteria*, *Firmicutes*) in the microbiome of oral cancer and pre-cancer patients.

### OTUs distinguishing cancer from anatomically matched contralateral normal patient samples

**Figure 4:**
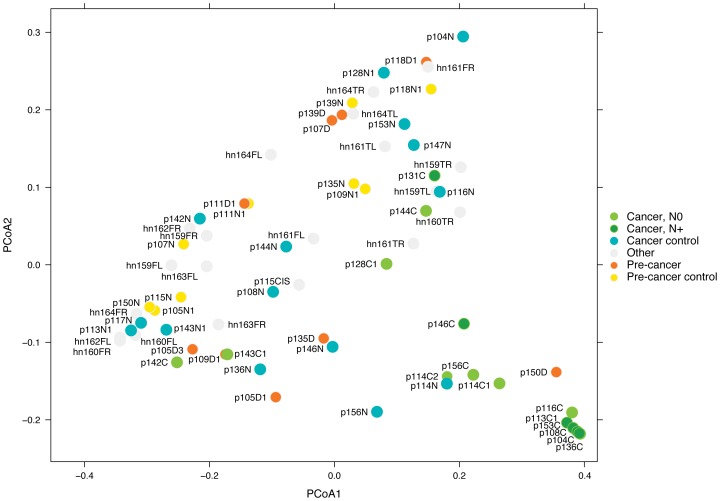
Distinguishing cancer and normal samples. PCoA based on Weighted UniFrac distance between samples given abundance of 12 OTUs. Axis 1 (PCoA1): 54% of variation explained. Axis 2 (PCoA2): 24% of variation explained. N0 and N+ indicate the nodal status of the cancer patient, N0  =  node negative, N+  =  node positive. Cancer control and pre-cancer control are contralateral clinically normal patient samples. Other identifies samples from healthy normal individuals.

To address the need for biomarkers to predict behavior of oral cancers that could be assayed by non-invasive tests, we asked whether cancer samples could be distinguished by bacterial composition. Considering cancer/normal paired samples, we identified 11 OTUs from the phyla *Actinobacteria* (*Actinomyces* and *Rothia*, 2 OTUs from each genus) and *Firmicutes* (*Streptococcus*, 7 OTUs) that were significantly decreased in cancers and one OTU from the phylum *Fusobacteria* (*Fusobacterium*) that was increased in cancers compared to anatomically matched contralateral normal patient samples (Table S17). Weighted UniFrac principal coordinates analysis (PCoA) based on this set of OTUs separated most cancers from normal and pre-cancer samples with five of the seven lymph node positive cases forming a tight cluster in the lower right corner of the plot (Figure 4). Metastasis to the cervical (neck) lymph nodes is a major determinant of oral cancer patient survival [35]. The current lack of reliable methods to assess risk of metastasis results in patients being routinely subjected to additional surgery to remove the lymph nodes, even though the majority will not benefit from the procedure. The tight clustering of samples from node positive patients in Figure 4 suggests that shifts in the composition of the oral cancer microbiome may also hold promise as a tumor associated biomarker of risk of metastasis.

### Whole micrbiome – β diversity

To investigate sample-to-sample dissimilarity, we subsampled the data by randomly selecting 31,109 sequences from each community to adjust for variation in sequencing depth (Table S18). When considering the three sample groups (Cancers, Healthy Normals, and Pre-cancers), we observed significant microbiome differences for patient identity based on both weighted UniFrac (abundance) and Unweighted UniFrac (presence/absence) metrics (Table S19, Figures S7 and S8). No other comparisons, such as left vs. right, number of sequences or lesion (cancer or pre-cancer) vs. control normal sites revealed significant differences. These observations are consistent with other studies that have highlighted the inter-individual differences in the oral microbiome [24]. Further, they support our study design, which measures changes in the microbiome within individuals, *i.e*., using each patient as his or her own control.

## Discussion

To study oral malignancy-associated microbiome changes, we performed a Discovery screen (Study 1), in which we non-invasively sampled cancers and contralateral clinically normal tissue samples from each individual. Comparison of the composition of the microbial communities within patients identified changes in abundance of *Actinobacteria* and *Firmicutes*. We confirmed these observations in a second Confirmation Cohort (Study 2) and further found significant changes in the abundance of the *Actinobacteria* genus *Rothia* and the *Firmicutes* genus *Streptococcus* when considering all cancers in Study 2 (Table S16). Although we did not see a significant change in abundance of the phylum *Fusobacteria* in either the Discovery or Confirmation Cohorts, we did find a significant increase in abundance of the *Fusobacteria* genus, *Fusobacterium* when considering all cancer patients in Study 2. We note that while the cohorts of patients studied here are small and heterogeneous, our findings regarding abundance of phyla are similar to published studies, which focused on comprehensive analysis of the oral microbiome. Moreover, the use of different sequencing technologies to measure the abundance of 16S rDNA amplicons in Studies 1 and 2 supports the robustness of our observations. Nevertheless, further larger studies should help to better define the oral cancer associated changes in abundance of these phyla and genera. Moreover, we observed changes in abundance of *Firmicutes* (*Streptococcus*) in association with oral pre-cancers, suggesting that oral lesion associated shifts in the composition of the microbial community may occur early in oral cancer development and/or herald cancer progression.

Smoking is a risk factor for oral diseases, including cancer and periodontitis. Studies have established that smoking impacts the composition of the bacterial communities in the oral cavity, including, for example, the salivary microbiome of healthy smokers and non-smokers [36] and the subgingival microbiomes of patients with periodontal disease [37], as well as the formation of plaque biofilms [38]. In our study, we found no evidence of overall differences in the microbiomes that could be attributed to smoking, but with only three current smokers and four non-smokers in our cancer patient cohorts for example, we cannot draw any conclusions at this time. On the one hand, because we used each patient as his/her own control, we would not expect to see smoking associated differences in abundance of microbiota associated with cancer, since smoking would affect both the control site and the cancer. On the other hand, in smokers, cells at the clinically normal sites and the cancers my respond differently to smoking induced changes in biofilm formation, for example, raising the possibility that while cancer associated changes in the abundance of microbiota in smokers and non-smokers appear similar, the formation and functional consequences of the altered microbiomes may differ in these patient groups. Similar considerations could apply to immunosuppressed individuals.

The presence of bacteria in oral cancers and/or differences in the bacterial communities associated with oral cancer have been reported previously using either culture dependent [13], [14] or molecular methods [15], [16], [17], yet no consistent observations have been reported across these studies. It is, however, difficult to make comparisons even amongst two recent studies reporting abundance of bacteria [16], [17] and this study, because of differences in (a) sample type (swab, this study vs. tissue sample [16], [17]), (b) oral cavity site, (c) source of patient matched normal control samples (anatomically matched contralateral clinically normal, this study, upper aerodigestive tract mucosae [16], adjacent normal [17]), (d) amplified region of the 16S ribosomal gene (V4, this study, V4-V5 [16] or V1-V4 [17]), (e) methodology (Sanger [16], [17] vs. pyrosequencing or MiSeq, this study), and (f) number of clones or sequence reads assigned to OTUs per sample (average 8000 and 55,000 reads per sample, Study 1 and 2, respectively, compared to ∼90 or ∼250 clones per sample [16], [17]). For example, Pushalkar and colleagues [16] reporting on 10 patients found that 75 and 80% of clones (normal and cancer, respectively) were assigned to the phylum *Firmicutes*. This proportion is not only higher than the 40–0% reported in other studies of the oral cavity of healthy or cancer patients, but since only ∼90 clones were sequenced per sample, there are too few clones to reliably determine relative abundance of other phyla for comparisons. A phylum level analysis of only the 16 tongue, floor of mouth and oral cavity cancers reported by Bebek and colleagues [17], however, revealed cancer associated increase in abundance of the phylum *Fusobacteria*, consistent with our observations, but decreased abundance of *Streptococcus* could not be seen, as few clones were assigned to this genus.

We cannot distinguish whether the observed shifts in the microbial community reflect the fact that certain bacteria are more suited to adhere and grow in the cancer microenvironment or whether they are cancer promoting. Further, it is unclear how to weigh the potential contributions from changes in abundant genera such as *Streptococcus* compared to the less abundant *Actinobacteria* genera. Potential roles for bacteria and fungi in cancer promotion include generation of carcinogenic substances, such as nitrosamine or other pro-carcinogenic chemicals, chronic inflammation and direct effects on signaling in epithelial cells resulting in enhanced proliferation or suppression of apoptosis [6], [7], [12], [39]. Only a minority of the oral microbial community can adhere to hard and soft oral tissues, and assembly of the complex oral biofilm is accomplished by subsequent adherence of secondary colonizers. *Streptococcus* is an early colonizer and *Fusobacterium* (*e.g*., *F. nucleatum*) has a propensity for co-aggregation with many genera, forming a bridge between early and late colonizers in the oral biofilm [40]. Thus, on the one hand, the observed decrease in prevalence of *Streptococcus* and increased abundance of *Fusobacterium* genera in pre-cancers could reflect the altered surface properties of the cancer cells and stroma, which might no longer support adhesion of *streptococci*. On the other hand, we can hypothesize that shifts in abundance of these two genera could result in an enhanced pro-inflammatory environment, since *Streptococcus* species have been reported to attenuate *Fusobacterium nucleatum* induced pro-inflammatory responses of oral epithelial cells [41], [42]. We also note that *Fusobacterium nucleatum* grown as a biofilm is capable of invading organotypic cultures [43], and secondly, that the organism has recently been reported in colon cancers [44], [45], further supporting a potential role in oral cancer.

The oral cavity offers a relatively unique opportunity to screen at risk individuals for (oral) cancer, because the lesions can be seen, and as we report here, the shift in the microbiome of the cancer and pre-cancer lesions compared to anatomically matched clinically normal tissue from the same individual can be detected in non-invasively collected swab samples. Saliva is another non-invasively collected oral sample composed largely of bacterial cells, but also shed epithelial and immune cells. A variety of “omics” biomarkers in saliva have been proposed for use in diagnosis of oral cancer, including metabolites, proteins, transcribed genes, miRNAs, genome alterations and epigenomic changes, as well as the microbiome [16], [46], [47], [48], [49]. For the microbiome, however, saliva may not be optimal. Saliva bathes the entire oral cavity, resulting in a loss of information on the subsite specific composition of bacterial communities. Moreover, with saliva, there is no possibility to use each individual as his or her own control, and so account for the substantial variation in the oral microbiome amongst individuals.

Non-invasively sampling the microbiome of oral lesions and corresponding normal tissue opens the possibility to not only detect cancer-associated changes at one time point, but the relative stability of the adult oral microbiome [25], [28], [50] also offers the opportunity to monitor shifts in bacterial communities over time. Here we observed changes in the microbiome, which, in future larger studies, may be confirmed as a potential biomarker of oral cancers or pre-cancers, and may even have utility to discriminate patients with lymph node metastases (Figure 4). In addition, there are other challenges in clinical management of oral cancers and pre-cancers that would benefit from better diagnostic tools. Most oral cancers are preceded by oral epithelial dysplasia (pre-cancer), a lesion, which unpredictably transforms to cancer. Oral cancer patients are also at risk of second primary cancers and recurrences. The microbiome may provide signatures that can be used as a biomarker for (a) progression of pre-cancers to cancer, (b) distinguishing oral cancer subtypes, (c) monitoring field changes associated with the high rate of second primary oral cancers and recurrences, and (d) predicting clinical behavior such as metastasis (Figure 4). We also highlight the possibility of medically modulating the oral microbiome for treatment of oral pre-cancers and damaged fields (field cancerization).

## Methods

### Ethics statement

The study was approved by the Institutional Review Board of New York University College of Dentistry and all patients provided written informed consent.

### Study population and biospecimen collection

In Study 1, samples from cancer and anatomically matched contralateral clinically normal regions of the oral cavity were obtained from five patients with oral cancer who were referred to the Bluestone Center for Clinical Research, New York University College of Dentistry in the period July 2011 to March 2012. Individuals enrolled in Study 2 included cancer and pre-cancer patients who were referred to the Bluestone Center for Clinical Research during the period April 2011 to August 2012 and individuals with no history of oral cancer (healthy normal). For this study, we used a well-defined clinical protocol for swabbing the oral lesion and a contralateral normal site. In addition to sampling the microbiome, the procedure provides a tumor genomic DNA sample with a DNA copy number profile that, when tested, reflects the profile obtained with the genomic DNA isolated from an incisional biopsy of the lesion; however, the procedure is not designed to optimally sample the entire oral microbiome.

Specifically, to collect samples from the cancer or pre-cancer, the lesion was dried by blotting with gauze and then the lesion was stroked with an Isohelix SK-2 swab (Cell Projects Ltd., Harrietsham, UK). The swab was held at an angle of approximately 20° relative to the surface of the lesion and one side of the swab was stroked across the lesion 10 times applying gentle downward pressure. The swab was then rotated 180° and the other side of the swab was stroked 10 times across the lesion in the same manner. Anatomically matched contralateral normal tissue and tissues from healthy normal individuals were sampled using an Isohelix swab, the brush from the OralCDx Brush Test (CDx Diagnostics, Suffern, NY) or a Rovers Orcellex brush (Rovers Medical Devices B.V., Oss, The Netherlands). The swabs and the brushes provided with the OralCDX Brush Test were placed into the tube provided as part of the Isohelix SK-2 swab or a microfuge tube, respectively and kept on ice for no more than 30 minutes prior to adding the Isohelix cell lysis and DNA stabilization solution (LS solution, 500 µL) and proteinase K solution (20 µL) both provided in the Isohelix DSK-2 kit. Samples were stored at room temperature and subsequently shipped to the University of California San Francisco for nucleic acid extraction.

To assess the reproducibility of the swab procedure, we included six pairs of replicate swabs taken at the same visit as the original swab as part of Study 2 (Tables S6 and S13). Five pairs of samples showed good concordance (Pearson correlation, minimum  = 0.628, first quartile  = 0.801, median  = 0.868, mean  = 0.843, third quartile  = 0.912, maximum  = 0.962), indicating that in most cases the procedure reproducibly samples the microbial communities of oral lesions and normal sites. Sample p113N2, however, showed little concordance with p113N1 (R^2^  = 0.116), but good concordance with p113C2 (R^2^  = 0.702). In other analyses, p113N2 behaved similarly to the cancer samples from patient 113 (data not shown), suggesting an error had occurred at some point during collection and processing of this sample.

### DNA extraction

Swabs in Isohelix tubes with 500 µL solution were vortexed, then centrifuged briefly and the liquid removed to a fresh 1.5 mL microfuge tube. This process was repeated 2–3 more times before transferring the swab to a fresh 1.5 mL microfuge tube and centrifuging at 14,000 rpm for 1 minute to extract the remaining cell lysate from the swab. The DNeasy blood and tissue kit (Qiagen Corp.) was used to extract nucleic acid from the solution recovered from the swab, following the manufacturer's protocol and including an initial incubation with Proteinase K (addition of 20 µL solution provided as part of the DNeasy blood and tissue kit) at 56°C for 10 minutes. The DNA concentration was determined using the Qubit v2.0 fluorometer.

### 16S rRNA amplicon preparation and 454 pyrosequencing

The V4 region of the small subunit ribosomal RNA gene (16S rRNA) was selected for study, because it is suited to analysis on multiple high throughput platforms yielding short reads. It has been reported to give low error rates when assigning taxonomy [51], [52] and to be suitable for community clustering [53]. For Study 1, the region was amplified using the primer set 515F (5′-GTGCCAGCMGCCGCGGTAA-3′) and 806R (5′-GGACTACVSGGGTATCTAAT-3′) [32], [54]. The complete forward primer construct (5′-3′) consisted of the Roche 454 Life Sciences Sequencing FLX Adaptor A (Roche Applied Science, Branford, CT, USA), a 12 bp Golay nucleotide barcode, and a GT linker followed by the 515F primer sequence. The 806R was similarly constructed but incorporated the Roche 454 Life Sciences Sequencing FLX Adaptor B, and a GC linker followed by the 806R primer sequence.

For each patient sample in Study 1, three separate amplifications were carried out in 25 µL reaction volumes. Each reaction contained 1 7L each of forward and reverse primers at a 10 µM concentration, 10 µL of 5 Prime HotMasterMix (5 Prime Inc., Gaithersburg, MD, USA), and 1 µL of extracted genomic DNA from each patient sample (concentration range  = 2.3–64.2 ng/ µL). After denaturation at 94°C for 3 minutes, 35 cycles of incubation at 94°C for 45 seconds, 50°C for 30 seconds, and 72°C for 1.5 minutes were carried out, followed by a 10 minute final elongation step at 72°C. The replicate reactions from each patient sample were pooled together by sample and quantified using the Quant-IT PicoGreen dsDNA Assay (Invitrogen, Carlsbad, CA, USA). Barcoded samples were then normalized to equimolar amounts to ensure equal sequencing depth for each sample, and finally pooled into one combined sample, which was further purified using the UltraClean PCR Clean-Up Kit (Mo Bio Laboratories, Inc., Carlsbad, CA). A final quantification was performed using the Quant-IT PicoGreen dsDNA Assay. Pooled amplicon libraries were sequenced unidirectionally in a single run on the Roche GS Junior instrument (100,000 reads per run) using the A beads for emulsion PCR.

For Study 2, the 16S V4 region was amplified using 515F and 806R fusion primers tailed with sequences to incorporate Illumina flow cell adapters with indexing barcodes [32]. Each sample was PCR amplified in a 10 µL reaction. The reaction contained 1 µL of a mixture of forward and reverse primers at a 5 µM concentration, 5 µL of Qiagen HotStar Master Mix (Qiagen Inc., Gaithersburg, MD, USA), 0.5 µL genomic DNA from each patient sample (20–50 ng) and nuclease free water. After incubation at 94°C for 15 minutes, 35 cycles of incubation at 94°C for 30 seconds, 50°C for 30 seconds and 72°C for 30 seconds were carried out, followed by incubation at 72°C for 10 minutes and storage at 4°C. Primer dimers and low molecular weight products were removed using Agencourt Ampure Beads (Beckman Coulter, Inc., Indianapolis, IN) and samples were quantified and quality checked for amplicon size using the Agilent Bioanalyzer. Amplicons (1×10^10^ molecules) were pooled. The pooled sample was diluted to 3.5 pM and 8 pM phiX DNA was spiked in to a final concentration of 2 pM. Amplicons were sequenced from both ends for 250 cycles using primers designed for paired-end sequencing avoiding the PCR amplification primers. The indexing barcode was sequenced using a third sequencing primer and an additional 13 cycles. Sequence data are available at the European Bioinformatics Institute, accession number PRJEB4953.

### Data analysis

We used QIIME [55] and custom scripts to process the sequencing data. Sequences were quality filtered and de-multiplexed using exact matches to the supplied DNA barcodes. Paired end sequencing performed on the MiSeq resulted in forward and reverse reads with some overlap in the V4 hypervariable region of the 16S. Each forward and reverse pair were stitched together using pandaseq with parameters "-F -l 220 -L 300 -t 0.5". Read pairs without sufficient overlap to allow stitching were discarded. For both MiSeq and 454 sequences, the resulting sequences were then searched against the Greengenes reference database of 16S sequences from 4 February 2011 [56], clustered at 97% by uclust. Sequences not matching any Greengenes reference sequence at >97% sequence identity were discarded (closed-reference OTU picking). The longest sequence from each Operational Taxonomic Unit (OTU) thus formed was then used as the OTU representative sequence, and assigned taxonomic classification using mothur's bayesian classifier, trained against the Greengenes reference database of 16S sequences from 4 February 2011 [56], clustered at 98% (bootstrap confidence level  = 80%). We note that ambiguous sequence identifications between species, for example, were addressed by not assigning any species identification to such reads, rather they were identified at broader levels (*e.g*., phylum, genus) where confident assignments could be made.

After pooling sequences into OTUs as described above, we generated a table listing the abundance of each OTU in each microbial community in this study (the OTU table). To obtain a summary statistic representing the overall dissimilarity between any two microbial communities in this study, we used the phylogenetically aware dissimilarity measures Unweighted UniFrac [57] and Weighted UniFrac [58]. Because some OTUs are closely related, while others are more distantly related, these two community-wide dissimilarity measures provide a more informative assessment of community resemblance than a more naive approach of *e.g*., counting shared OTUs between communities. Unweighted UniFrac considers only presence/absence of taxa and counts the fraction of the branch length unique to either community. In weighted UniFrac, the branch distance is weighted by difference in abundance. In both cases, the result is a scalar measure of dissimilarity between each pair of communities in this study, and is based on the information contained in the OTU table as well as the phylogeny relating the OTUs. We used the supplied Greengenes phylogeny (which is a curated version of a FastTree constructed phylogeny built from full length Greengenes 16S/SSU sequences). Because these measures are sensitive to differences in sequencing depth amongst samples, we randomly selected 1,038 (Study 1) or 31,109 (Study 2) sequences from each sample (subsampled dataset), and generated the distance matrix by a pair-wise inter-comparison of the profiles in the subsampled dataset. We used PCoA to compare samples based on the Unweighted and Weighted UniFrac distances. We clustered samples by applying the average neighbor (HC-AN) method as implemented in hclust (R package http://www.R-project.org) to the distance matrix.

### Statistical analysis

Statistical tests with p-values less than 0.05 were considered significant. We used a binomial test to evaluate the significance of finding consistent changes in abundance of three phyla in the five Discovery cohort patients (Study 1). In the absence of any cancer effects, we expect that in a single patient, the chance that a microbial phylum would increase in the cancer sample is 0.5, as is the chance that the phylum would decrease. Although our counts are discrete, the probability of "no change in abundance at all" is so improbable that we can neglect it. Therefore we can calculate the probability that (all five patients show an increase or all five patients show a decrease) – a two-sided binomial with p  = 2*(1/2)∧5  = .0625. In our data, three of the five major phyla show such a unanimous shift (all five patients show an increase or all five patients show a decrease). The probability that (three or more phyla have such an unanimous shift) absent any cancer effects is thus the CDF of a binomial with B(n = 5,p = 0.0625) to x = 3. p = 0.0022 in this case.

The adonis function implemented in the vegan package [59] was used to test for whole microbiome differences between sample groups.

For comparisons of specific sample groups, we used the relative abundance of OTUs after normalizing samples to one million counts. The normalization was done only for ease of display – no imputation of missing sequences was performed. A paired t-test was performed to identify OTUs significantly increased or decreased across cancer samples relative to the normal samples from the same patient, and Welch's *t* test (a two tailed, unequal variance test) was used to identify OTUs significantly increased or decreased across samples from two groups of patients.

## Supporting Information

Figure S1 Rarefaction curves displaying average number of families and genera detected vs. sequencing depth in Study 1. For each point, sequences were subsampled without replacement 10 times and displayed is the average number of families (a) or genera (b) found. There is a fairly wide range of α diversity. For example, at 5,000 sequences per sample, the number of families detected ranges from ∼15–28.
(TIF)

Figure S2 Rarefaction curves displaying average number of OTUs detected vs. sequencing depth in Study 2. For each point, sequences were subsampled without replacement 10 times and displayed is the average number of OTUs found. Sample hn164FL has relatively high OTU diversity. Note, also that here we show diversity at the OTU level, whereas in Figure S1, the rarefaction curves are shown at the Family and Genus levels.
(TIF)

Figure S3 Data from studies 1 and 2 are highly correlated. (a) Scatterplots comparing sequence counts for OTUs determined in Study 1 (454, X-axis) with Study 2 (MiSeq, Y-axis). Shown is the Pearson correlation for each pair of samples. (b) Summary of correlations. The sequence counts were square root transformed to bring in the outliers prior to computing the Pearson and concordance correlation coefficients.
(TIF)

Figure S4 Relative abundance of phyla in paired samples in Study 2. Shown are the percent of OTUs corresponding to the five more abundant phyla in cancer and pre-cancer samples and their anatomically matched contralateral normal samples and left and right samples from healthy normal subjects.
(TIF)

Figure S5 Diversity of *Firmicutes*, *Fusobacteria*, *Bacteroidetes*, *Proteobacteria* and *Actinobacteria* genera in cancer, pre-cancers and healthy normal samples. Read counts normalized to one million counts are shown for the genera accounting for >10% of OTUs in more than 20% of samples for each phylum.
(TIF)

Figure S6 Change in relative abundance of genera in Study 2. Change in relative abundance of genera representing 10% of OTUs in more than 20% of samples. Shown is the difference in abundance of genera associated with cancers and pre-cancers compared to anatomically matched contralateral normal samples. For healthy normal samples, we compared left and right sides of the lateral tongue or floor of mouth.
(TIF)

Figure S7 Hierarchical clustering based on Weighted (left) and Unweighted (right) UniFrac for three sample types (Cancer and contralateral normal, healthy normal left/right and Pre-cancer and contralateral normal).
(TIF)

Figure S8 Whole microbiome PCoA based on Weighted (left) and Unweighted (right) UniFrac for three sample types (Cancer and contralateral normal, healthy normal left/right and Pre-cancer and contralateral normal). Significant microbiome differences were observed for patient identity.
(TIF)

Table S1 Discovery cohort clinical characteristics.
(XLSX)

Table S2 Study 2 cancer patients.
(XLSX)

Table S3 Study 2 Carcinoma in situ patient.
(XLSX)

Table S4 Study 2 pre-cancer patients.
(XLSX)

Table S5 Study 2 healthy normal individuals.
(XLSX)

Table S6 Replicate samples from cancer and pre-cancer patients.
(XLSX)

Table S7 Sequence reads and OTUs in five patient matched cancer and normal samples (Discovery cohort).
(XLSX)

Table S8 Study 1 OUT table.
(XLSX)

Table S9 Study 2 cancer patients sequence reads and OTUs.
(XLSX)

Table S10 Study 2 pre-cancer patients sequence reads and OTUs.
(XLSX)

Table S11 Study 2 healthy normal individuals sequence reads and OTUs.
(XLSX)

Table S12 Study 2 carcinoma in situ patient sequence reads and OTUs.
(XLSX)

Table S13 Study 2 sequence reads and OTUs for replicate samples.
(XLSX)

Table S14 Study 2 OTU table.
(XLSX)

Table S15 Study 2 OTU table normalized to one million counts.
(XLSX)

Table S16 Changes in abundance of genera from the five more abundant phyla.
(XLSX)

Table S17 OTUs discriminating cancer and matched normal samples.
(XLSX)

Table S18 Study 2 OTU table adjusted for variation in sequencing depth. Data were subsampled by randomly selecting 31,109 sequences from each community.
(XLSX)

Table S19 Whole microbiome analysis - β diversity.
(XLSX)
