# Correction: Changes in Abundance of Oral Microbiota Associated with Oral Cancer

**DOI:** 10.1371/journal.pone.0106297

**Published:** 2014-08-15

**Authors:** The PLOS ONE Staff

Muy-Teck Teh should not be listed in the author byline. The correct citation is: Schmidt BL, Kuczynski J, Bhattacharya A, Huey B, Corby PM, et al. (2014) Changes in Abundance of Oral Microbiota Associated with Oral Cancer. PLoS ONE 9(6): e98741. doi:10.1371/journal.pone.0098741
