# Supplementary figures and images for: Changes in Abundance of Oral Microbiota Associated with Oral Cancer
Source: PLoS One. 2014 Jun 2;9(6):e98741. doi: 10.1371/journal.pone.0098741 (PMC4041887; doi:10.1371/journal.pone.0098741)

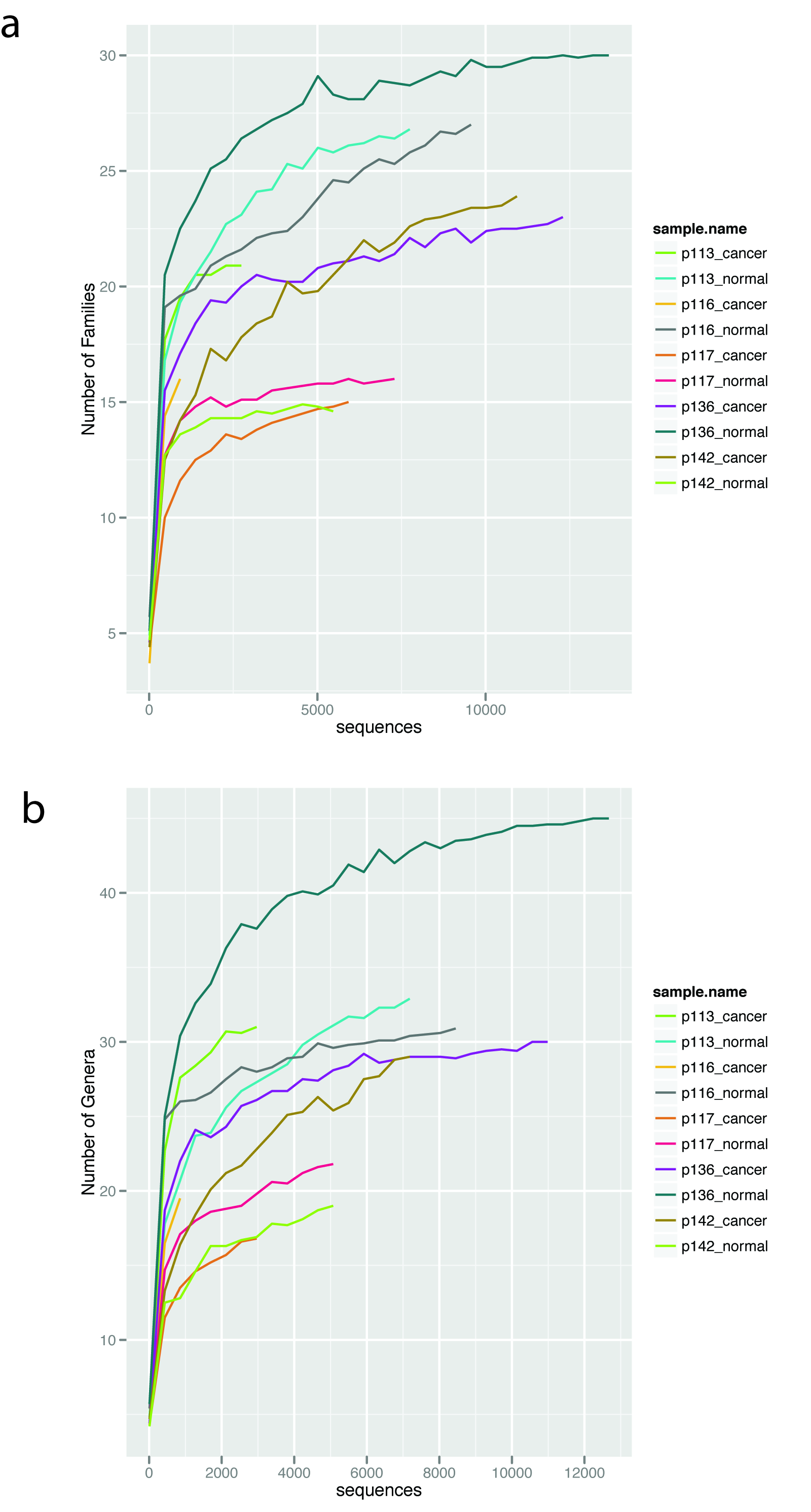

Supplement: Figure S1 — Rarefaction curves displaying average number of families and genera detected vs. sequencing depth in Study 1. For each point, sequences were subsampled without replacement 10 times and displayed is the average number of families (a) or genera (b) found. There is a fairly wide range of α diversity. For example, at 5,000 sequences per sample, the number of families detected ranges from ∼15–28. (TIF) [file pone.0098741.s001.tif]

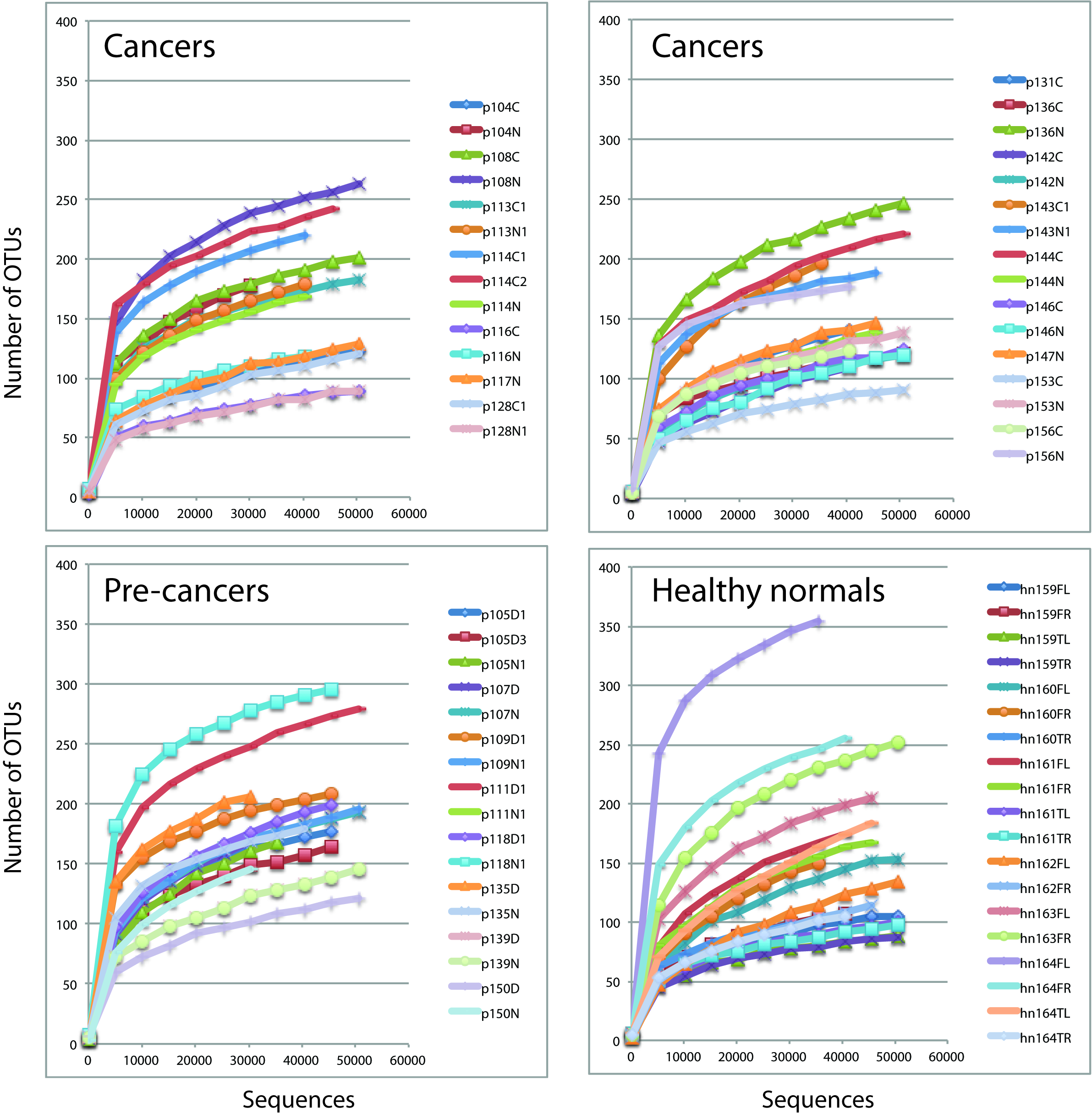

Supplement: Figure S2 — Rarefaction curves displaying average number of OTUs detected vs. sequencing depth in Study 2. For each point, sequences were subsampled without replacement 10 times and displayed is the average number of OTUs found. Sample hn164FL has relatively high OTU diversity. Note, also that here we show diversity at the OTU level, whereas in Figure S1, the rarefaction curves are shown at the Family and Genus levels. (TIF) [file pone.0098741.s002.tif]

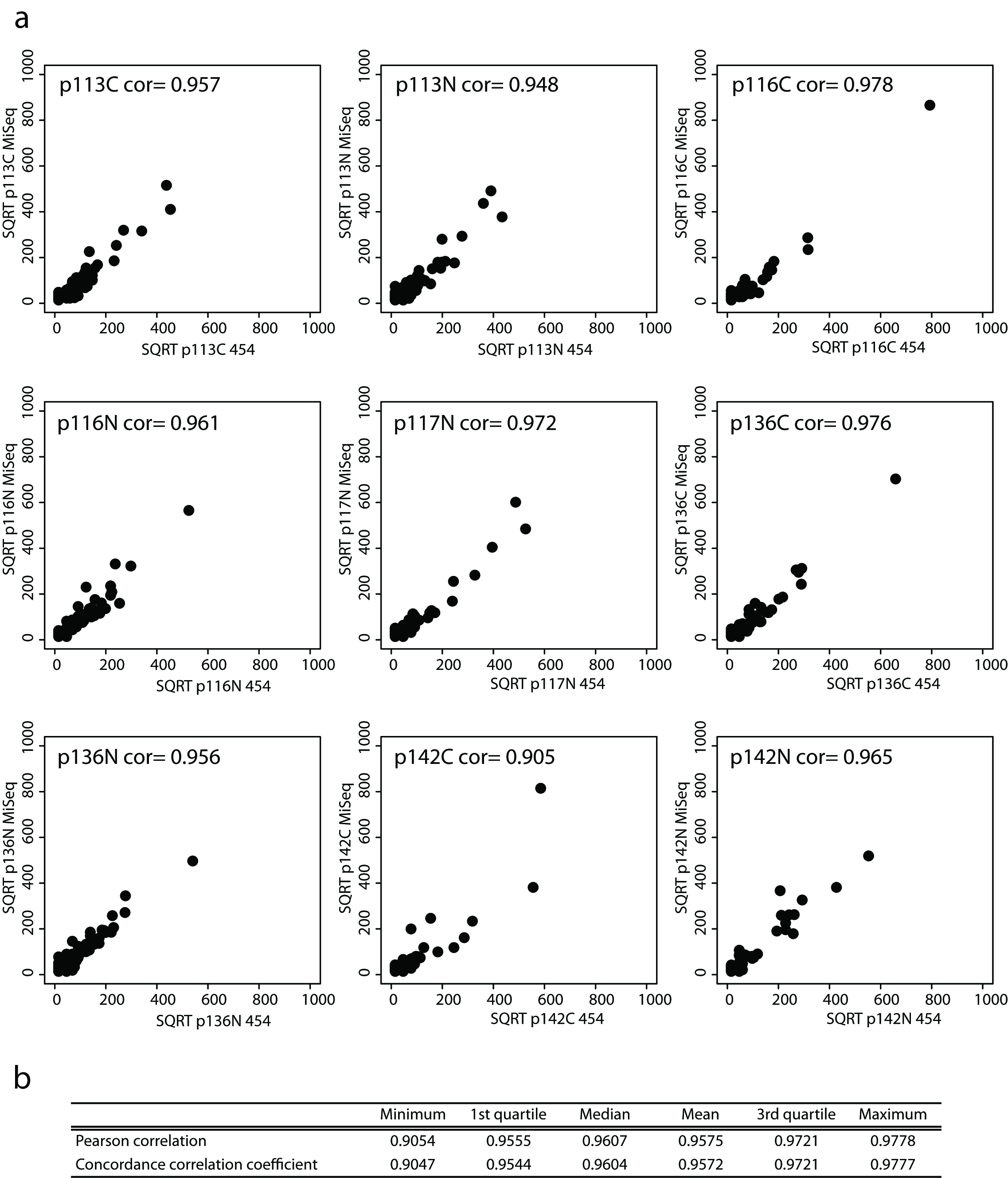

Supplement: Figure S3 — Data from studies 1 and 2 are highly correlated. (a) Scatterplots comparing sequence counts for OTUs determined in Study 1 (454, X-axis) with Study 2 (MiSeq, Y-axis). Shown is the Pearson correlation for each pair of samples. (b) Summary of correlations. The sequence counts were square root transformed to bring in the outliers prior to computing the Pearson and concordance correlation coefficients. (TIF) [file pone.0098741.s003.tif]

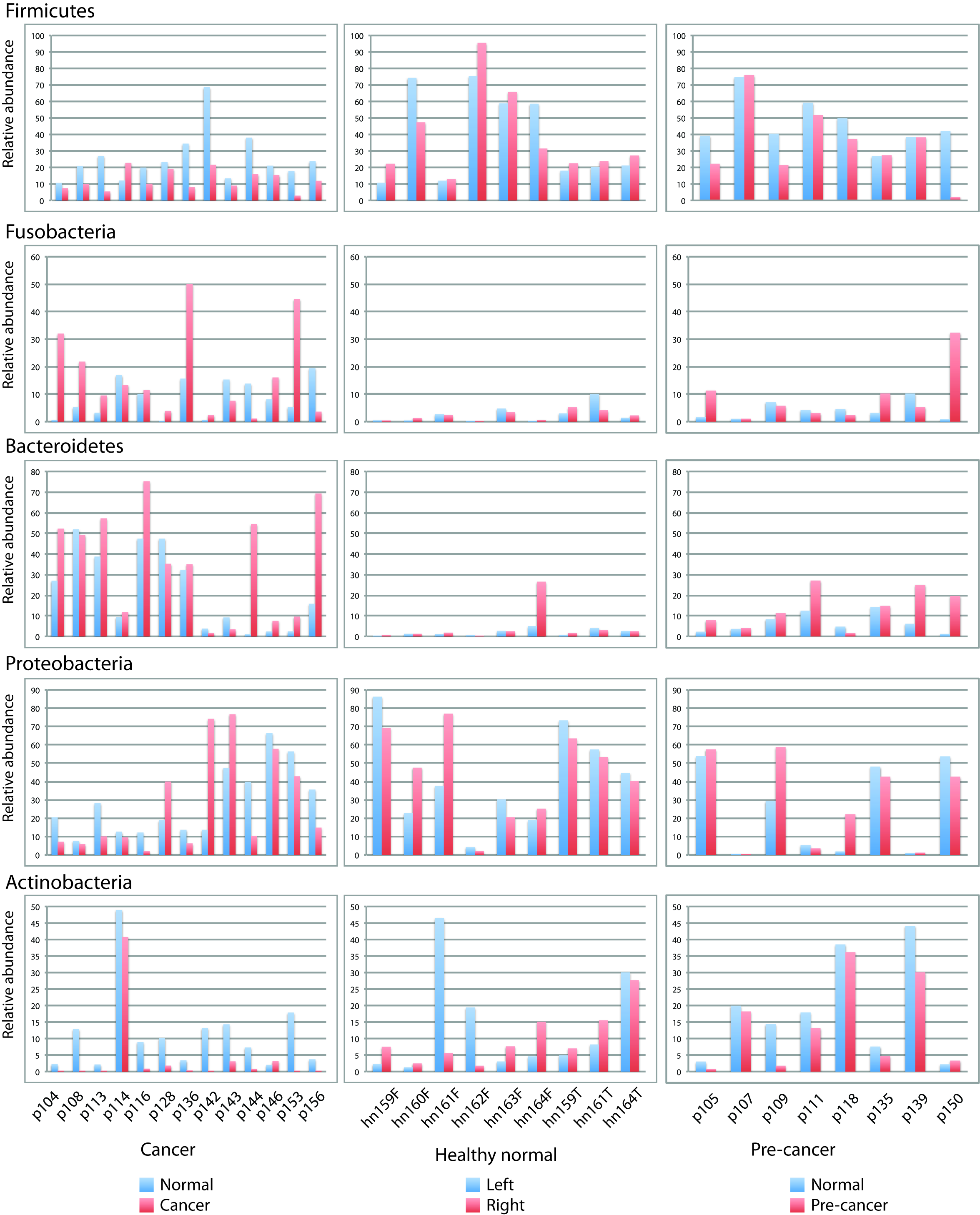

Supplement: Figure S4 — Relative abundance of phyla in paired samples in Study 2. Shown are the percent of OTUs corresponding to the five more abundant phyla in cancer and pre-cancer samples and their anatomically matched contralateral normal samples and left and right samples from healthy normal subjects. (TIF) [file pone.0098741.s004.tif]

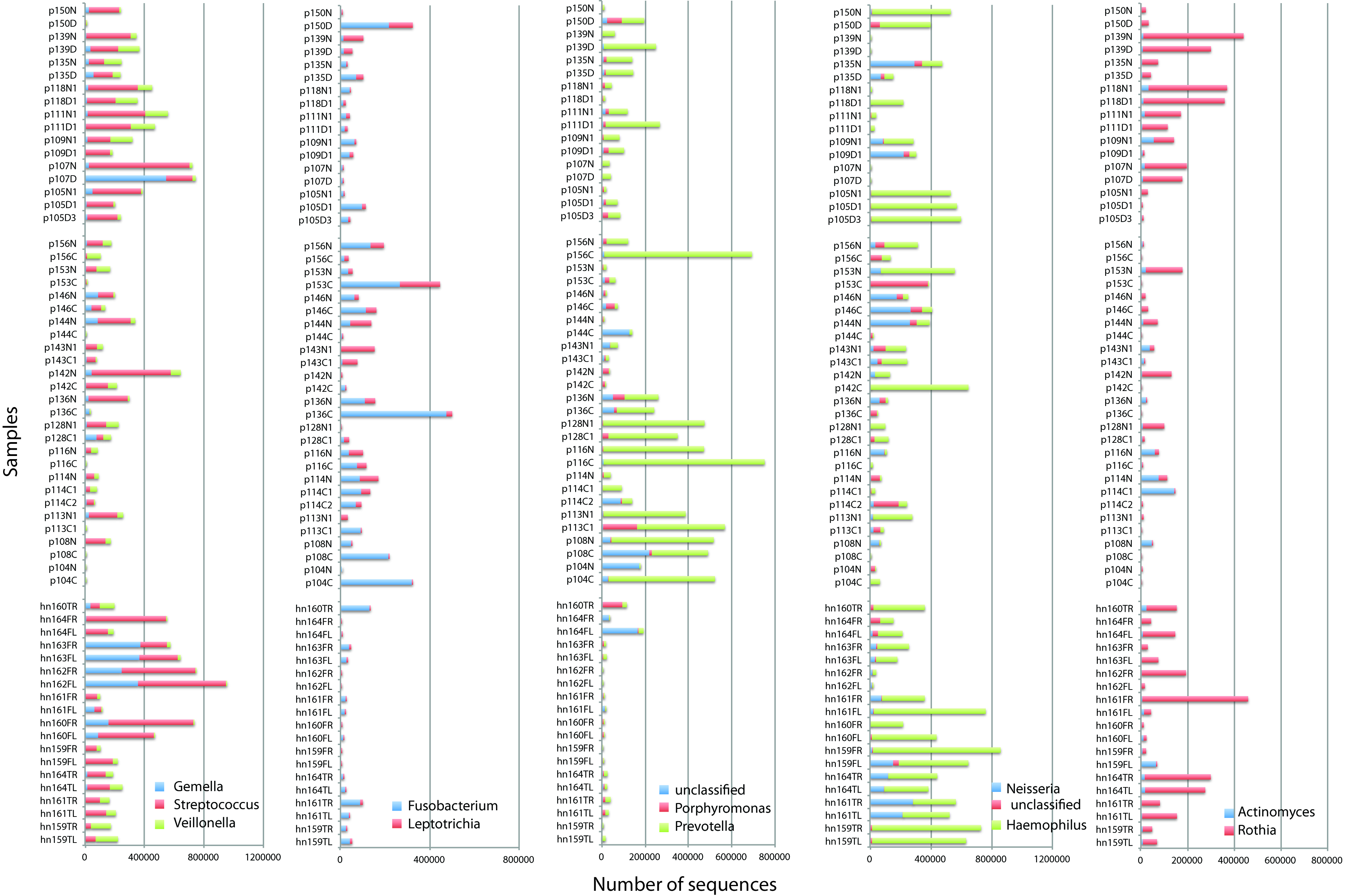

Supplement: Figure S5 — Diversity of Firmicutes, Fusobacteria, Bacteroidetes, Proteobacteria and Actinobacteria genera in cancer, pre-cancers and healthy normal samples. Read counts normalized to one million counts are shown for the genera accounting for >10% of OTUs in more than 20% of samples for each phylum. (TIF) [file pone.0098741.s005.tif]

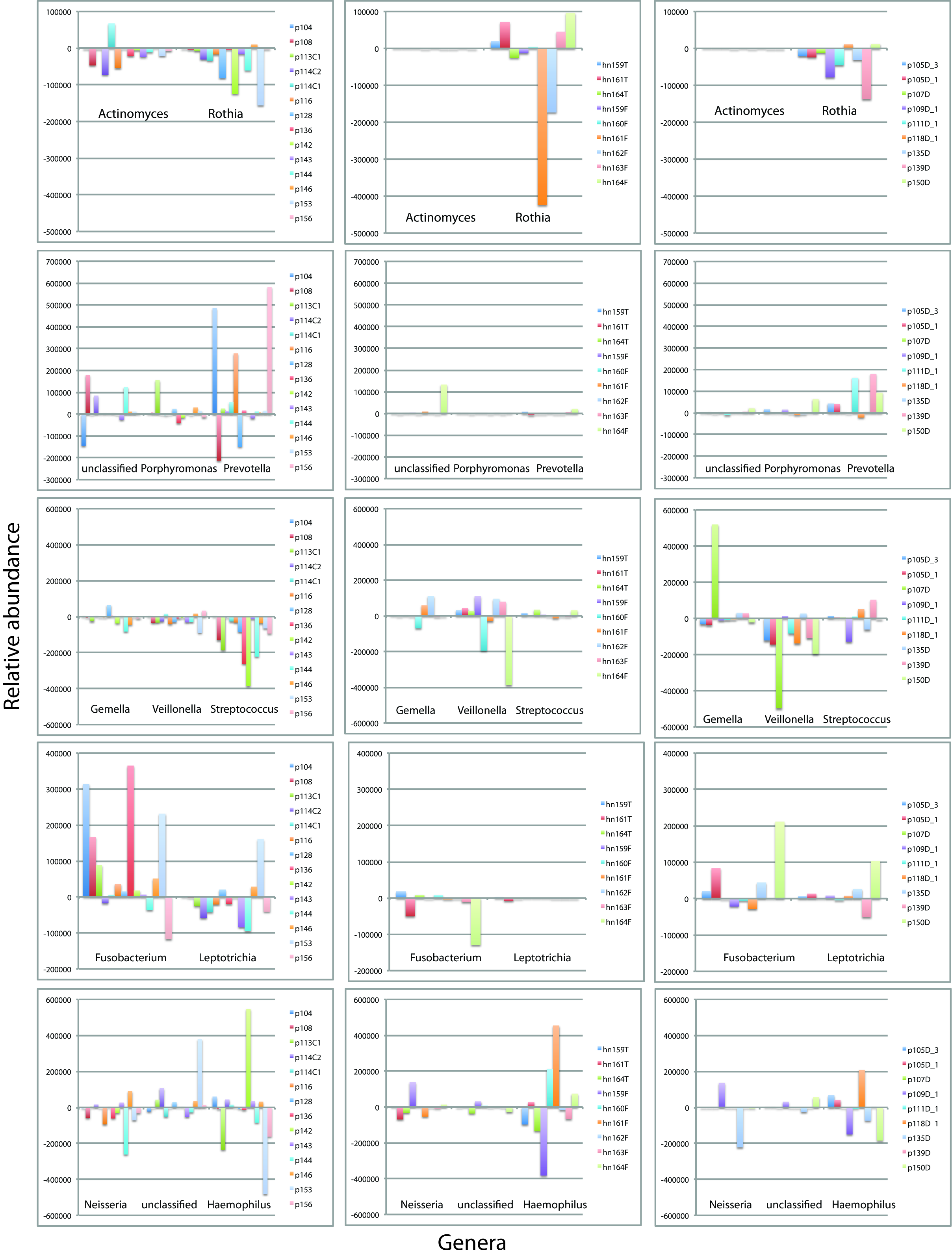

Supplement: Figure S6 — Change in relative abundance of genera in Study 2. Change in relative abundance of genera representing 10% of OTUs in more than 20% of samples. Shown is the difference in abundance of genera associated with cancers and pre-cancers compared to anatomically matched contralateral normal samples. For healthy normal samples, we compared left and right sides of the lateral tongue or floor of mouth. (TIF) [file pone.0098741.s006.tif]

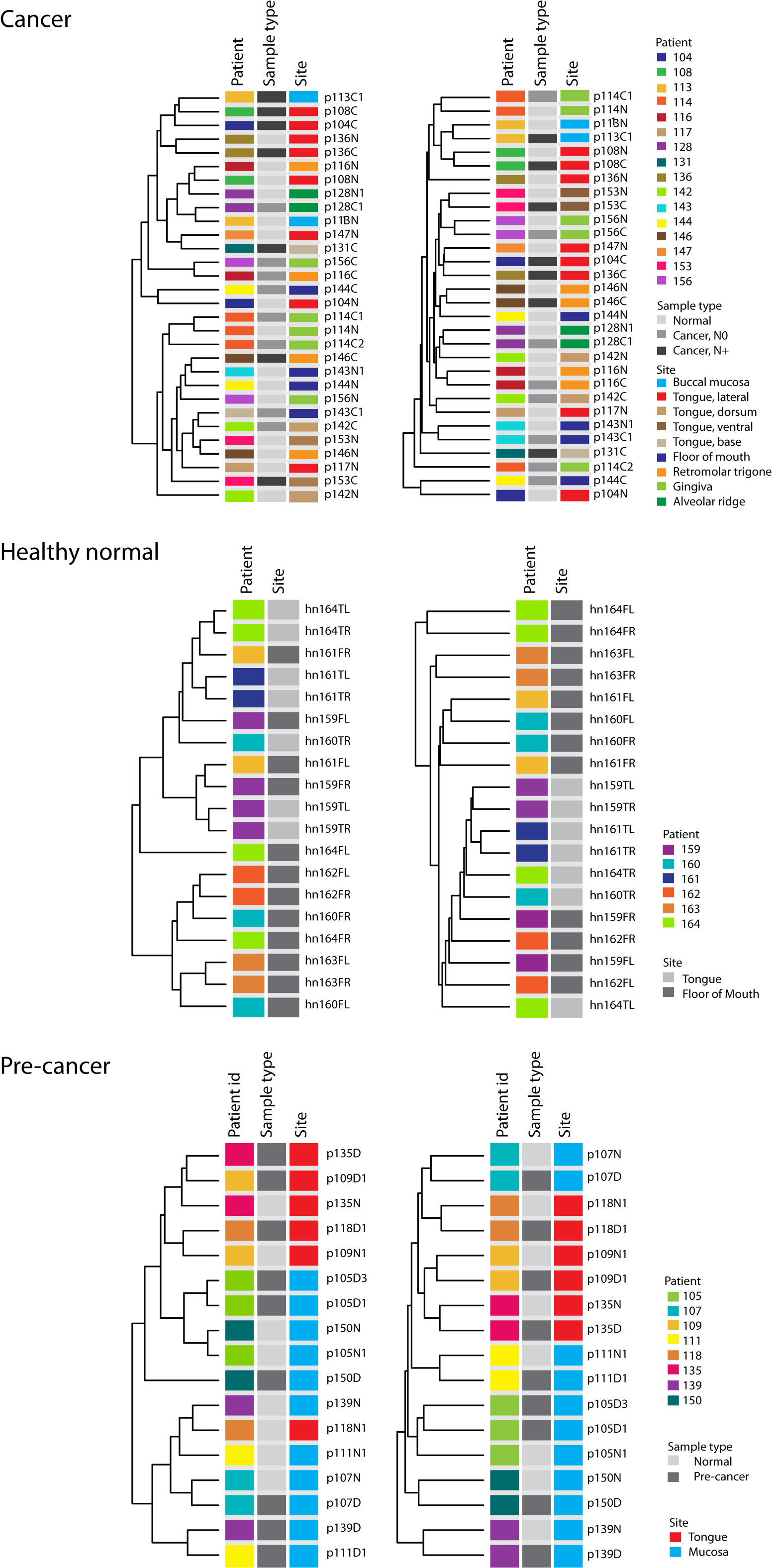

Supplement: Figure S7 — Hierarchical clustering based on Weighted (left) and Unweighted (right) UniFrac for three sample types (Cancer and contralateral normal, healthy normal left/right and Pre-cancer and contralateral normal). (TIF) [file pone.0098741.s007.tif]

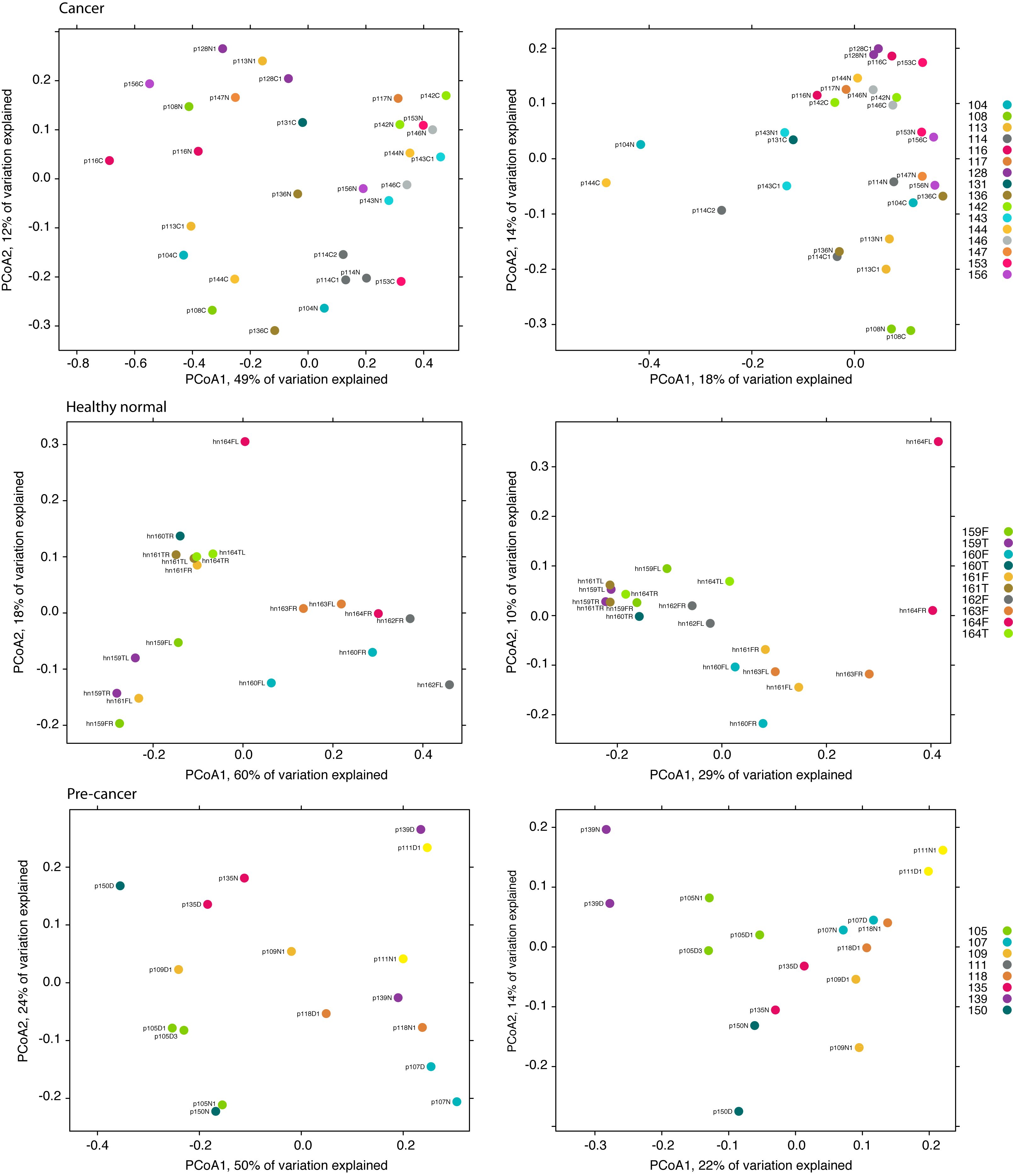

Supplement: Figure S8 — Whole microbiome PCoA based on Weighted (left) and Unweighted (right) UniFrac for three sample types (Cancer and contralateral normal, healthy normal left/right and Pre-cancer and contralateral normal). Significant microbiome differences were observed for patient identity. (TIF) [file pone.0098741.s008.tif]
